# Application of multiple testing procedures for identifying relevant comorbidities, from a large set, in traumatic brain injury for research applications utilizing big health-administrative data

**DOI:** 10.3389/fdata.2022.793606

**Published:** 2022-09-28

**Authors:** Sayantee Jana, Mitchell Sutton, Tatyana Mollayeva, Vincy Chan, Angela Colantonio, Michael David Escobar

**Affiliations:** ^1^Department of Mathematics, Indian Institute of Technology, Hyderabad, India; ^2^Toronto Western Hospital, Toronto, ON, Canada; ^3^Dalla Lana School of Public Health, University of Toronto, Toronto, ON, Canada; ^4^KITE Research Institute Toronto Rehabilitation Institute, University Health Network, Toronto, ON, Canada; ^5^Rehabilitation Sciences Institute, Temerty Faculty of Medicine, University of Toronto, Toronto, ON, Canada; ^6^Global Brain Health Institute, Institute of Neuroscience, Trinity College Dublin, Dublin, Ireland; ^7^Acquired Brain Injury Research Lab, University of Toronto, Toronto, ON, Canada; ^8^Institute of Health Policy, Management and Evaluation, University of Toronto, Toronto, ON, Canada; ^9^Faculty of Health Sciences, Ontario Tech University, Oshawa, ON, Canada; ^10^ICES (fomerly Institute for Clinical Evaluative Sciences), Toronto, ON, Canada

**Keywords:** Benjamini-Hochberg, Benjamini-Yekutieli, McNemar test, ICD-10 codes, health-administrative data

## Abstract

**Background:**

Multiple testing procedures (MTP) are gaining increasing popularity in various fields of biostatistics, especially in statistical genetics. However, in injury surveillance research utilizing the growing amount and complexity of health-administrative data encoded in the International Statistical Classification of Diseases and Related Health Problems 10th Revision (ICD-10), few studies involve MTP and discuss their applications and challenges.

**Objective:**

We aimed to apply MTP in the population-wide context of comorbidity preceding traumatic brain injury (TBI), one of the most disabling injuries, to find a subset of comorbidity that can be targeted in primary injury prevention.

**Methods:**

In total, 2,600 ICD-10 codes were used to assess the associations between TBI and comorbidity, with 235,003 TBI patients, on a matched data set of patients without TBI. McNemar tests were conducted on each 2,600 ICD-10 code, and appropriate multiple testing adjustments were applied using the Benjamini-Yekutieli procedure. To study the magnitude and direction of associations, odds ratios with 95% confidence intervals were constructed.

**Results:**

Benjamini-Yekutieli procedure captured 684 ICD-10 codes, out of 2,600, as codes positively associated with a TBI event, reducing the effective number of codes for subsequent analysis and comprehension.

**Conclusion:**

Our results illustrate the utility of MTP for data mining and dimension reduction in TBI research utilizing big health-administrative data to support injury surveillance research and generate ideas for injury prevention.

## Introduction

Biological functions are interrelated since they co-occur in the human body. This gives rise to complex relationships between different body functions and the effects of multiple diseases on the human body; therefore, it is essential to consider multiple coexisting medical disorders referred to as comorbidities (Feinstein, 1970) in this article and their implications in injury surveillance. Of all injuries known to date, traumatic brain injury (TBI) is among the most disabling injuries affecting many individuals in the prime of their life (Feigin et al., 2021). Concern about TBI related to the expansion of industrialization and armed conflict has led to increased interest in the epidemiology of TBI in civilians and among service members. Published estimates of TBI vary worldwide; although when estimates from studies with comprehensive data collection methods are extrapolated internationally, reports suggest that 50–60 million people are affected annually, and the pooled international incidence rate of TBI (excluding TBI with no overt pathologic features) is reported to be a staggering 349 (95% confidence interval (CI) 96–1,266) per 100,000 person-years. To develop prevention initiatives and guide injury surveillance research, it is necessary to consider the multiple comorbidities occurring in the time preceding injury in the population of interest.

Population-based health-administrative data housing information across multiple diagnostic conditions from millions of patients have become a popular data source for evaluating relationships between comorbidities with a specific condition of interest. Due to rapid advancement in technology and the evolution of computation and storage facilities over the last few decades, recording and accessing information across millions of study units in large electronic databases have become feasible, giving rise to “high-dimensional” or “big” data. There is a need to develop appropriate data mining and dimension reduction methods for managing big data efficiently. This change in thinking mirrors the change in the analyses of genetic data.

Initially, researchers encountered only a small group of genes in laboratory studies. However, it is now common for genetic studies to simultaneously analyse millions or trillions of genes (Thomas et al., 2005) in multiple testing (MT) procedure (MTP) that involves the simultaneous testing of more than one hypothesis. One strategy used by genetic researchers has been to look at the false discovery rate (FDR) (Tsai et al., 2003), which looks at a set of variables that have a high probability of having a “signal”. Instead of seeing if one gene is, say, statistically significant, the goal is to find a set of genes where there is a high probability that most of the genes are significant. This promising approach has yet to be widely used in analyzing healthcare data from large administrative databases of injury data of patients with TBI.

To control for family-wise error rate (FWER) in MTPs, the most common method was Bonferroni correction (Dunn, 1961). This is an adjustment so that the possibility of falsely rejecting the null hypothesis for each of the multiple tests is held at α. For example, for the usual α of 0.05, the chance of falsely rejecting the null hypothesis for all tests combined is 0.05, not for each individual test. For Bonferroni, one would require the probability of falsely rejecting each individual test to be fixed at α/*m*, where *m* is the total number of tests. However, in most genetic experiments and biomedical and epidemiological studies, scientists are generally interested in detecting true signals rather than just guarding against a large number of false positives by controlling FWER. In their seminal paper in 1995, Benjamini and Hochberg suggested an alternative approach for dealing with multiple tests, which has increased power, and is less conservative (Reiner et al., 2003; Tsai et al., 2003; Benjamini et al., 2006; Narum, 2006; Sun et al., 2006; Jones et al., 2008; Verhoeven et al., 2017). The authors (Benajmini and Hochberg, 1995) suggested controlling FDR, which is the expected proportion of false discoveries, a discovery being a rejected hypothesis or in other words a “signal”.

In this study, we provided an explanation and an illustration of how to utilize these FDR control methods to support injury surveillance research in TBI. Determining the significant association of multiple comorbidities with the TBI event requires us to test multiple hypotheses, which increases the chances of inferring false-positive results, and this rate accelerates with the number of hypotheses. This brings us to the domain of MT theory, which provides a mechanism to protect against false positive conclusions by controlling for error rate (Bender and Lange, 2001; Reiner et al., 2003). Although MTPs are gaining popularity in genetics literature (Tsai et al., 2003; Sun et al., 2006) and clinical trials (Marshall et al., 2004; Mehrotra and Heyse, 2004; Burkom et al., 2005; Mehrotra and Adewale, 2012), it is, however, underutilized in biomedical and epidemiologic research until recently by a few (Jones et al., 2008; Anderson et al., 2016; Sollmann et al., 2018, 2019), even though it is a common problem in this field (Bender and Lange, 2001). Before further discussion, it is essential to point out to readers that we will not be looking at complex interactions between comorbidities; we will simply be looking at distinct associations of comorbidities with the condition of interest, TBI.

The objective of this study is to demonstrate the use of contemporary methodologies of MTPs for accurate statistical analysis in big health-administrative data to capture distinct comorbidities associated with TBI. This article outlines the procedures, using a case study, so that researchers could potentially use while working with multiple comorbidities in health-administrative data of other complex injuries and conditions. In other words, this article demonstrates the use of modern data mining methods for handling big data from healthcare settings. The intent of this article is pedagogical, and we used knowledge translations and draw inspirations from other fields of big data such as statistical genetics (Tsai et al., 2003; Sun et al., 2006). Table 1 presents the applications of different MTPs across various domains.

**Table 1:** Applications of MTPs in different domains of applications.

**Reference**	**Domain**	**Objective**	**MTP used**
Bartenschlager and Brunner (2021)	Business applications	Develop methodology for FDR control	Bonferroni, Benjamini and Hochberg, Holm's, Benjamini and Yekutieli
Alberton et al. (2020)	Brain imaging studies	Multiple testing correction on multiple contrasts of parameter estimates	Bonferroni, Dunn-Sidak, Westfall-Young, Scheffe
Carvajal-Rodríguez (2018)	Genomics	Develop tool for multiple testing corrections	Holm's, Benjamini and Hochberg, Bon-EV,

## Methods

The methods mentioned below are guided by the scientific question to perform an MTP on a matched sample of TBI and non-TBI patients, enriched with complementary information of previous TBI research. We first discussed the development of required steps that can be easily translated for any similar big administrative healthcare data and then discussed the results in the light of previous TBI research. We would like to emphasize again that our objective in this article is simply pedagogical. Similar approaches already exist in statistical genetics literature, and we intended to translate that knowledge to the healthcare field. Details of the underlying MTPs have been provided in Appendix.

### Identification of data analytics methods and steps

In large health-administrative data sets, one way of classifying health conditions and their circumstances is to use the International Statistical Classification of Diseases and Related Health Problems, Tenth Revision (ICD-10) codes (Walker et al., 2012). Before using MTPs, we found that the complete set of ICD-10 codes was too granular, so we first grouped the codes by the first three characters – the first character being a letter and the next two being digits. Individual codes are nested into these groups, reducing the problem to only looking at a possible 2,600 codes.

**Figure 1:**
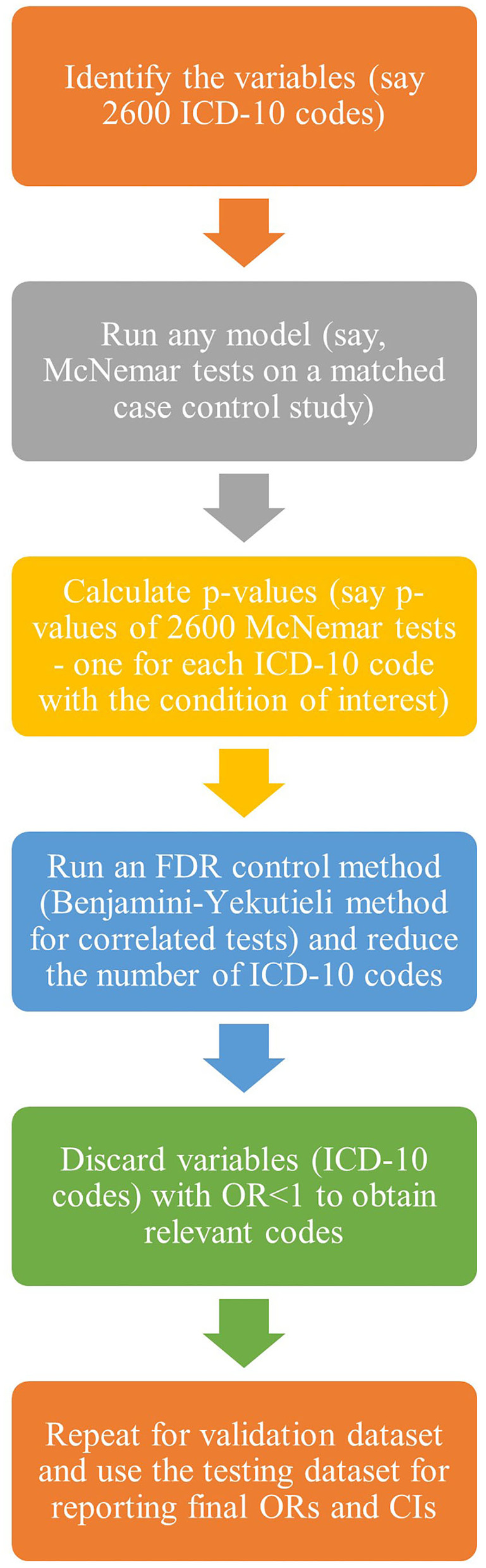
A diagrammatic flowchart for the steps.

Therefore, with techniques to fully exploit the potential wealth of information in health-administrative data and data reduction tools, we considered 2,600 ICD-10 codes for an aggregative association study of different comorbidities on a particular condition of interest, thus leading to 2,600 multiple tests. We elaborated on the application of MT theory for health-administrative data in the context of associations between 2,600 ICD-10 codes and TBI. Below are the basic steps of the analysis (Figure 1). The objective is to identify a subset of variables with a high probability of being related to the outcome of interest.

*Step 1*: Define the set of variables one wishes to test. In the example below, the purpose is to look at which ICD-10 codes appear to be related to a future TBI event.
*Step 2:* Decide the basic analytical model for each of the 2,600 variables being tested. The investigator can use almost any model in this study and obtain a *p*-value as a measure of the significance of the variable in the model. In this study, a matched case-control study was used; hence, McNemar test statistics were calculated.
*Step 3*: Repeat the test for each variable using the basic analytical model from step 2. In each analysis, the *p*-value is obtained. MTP is then used to identify a small set of *p*-values, controlling for FDR. In the example that follows, Benjamini and Yekutieli (2001) approach is used to account for possible dependence among the 2,600 tests to determine significant comorbidities.

### Proof-of-concept and internal validation

We used health-administrative data on emergency departments extracted from National Ambulatory Care Reporting System (NACRS) database and acute care data extracted from Discharge Abstract Database (DAD). Both data sets were obtained from ICES, which collects and stores health administrative data on publicly funded services provided to residents of Ontario, Canada. Information on the study subjects' income quintile was extracted from the Registered Persons Database.

We constructed a histogram for the days from the index date of hospital visits for all the TBI patients included in the study. We observed a peak around the index date, with the frequency dropping to a stationary point at 30 days before, and after, the index date. Therefore, we used this 60-day window as a TBI-related window, where we considered, all ED and acute care visits within 5 years, between the fiscal years 2007/08 and 2015/16, up to 30 days prior to a TBI event as the pre-injury phase. For further details on these and the histogram, we refer the readers to the follow-up study (Mollayeva et al., 2019).

We split the data into three groups, namely, training sample, validation sample, and test sample when doing this analysis. The advice on percentages of the data to put into each group varies. In this study, the master data were split into 50–25–25% of the data for train/validate/test data sets (Friedman et al., 2008). The analysis was first done on the training sample to obtain relevant codes, then retested using the validation data set. Relevant codes were reconfirmed using the validation data set. The testing data set was used for reporting the final output. This approach provided independent data sets to replicate the findings and to further guard against overfitting.

A 1:1 match was performed among the two groups being compared in this study, namely, TBI patients and non-TBI patients, or in other words, patients who were also discharged from ED or acute care during the same time period for a reason other than TBI. They were matched based on four demographic variables, namely, age, sex, income, and rural or urban neighborhood, using exact matching for sex, income quintile, neighborhood, and caliper matching for age with a caliper of 2 years. For additional details, the readers are referred to Mollayeva et al. (2019).

## Results

**Figure 2:**
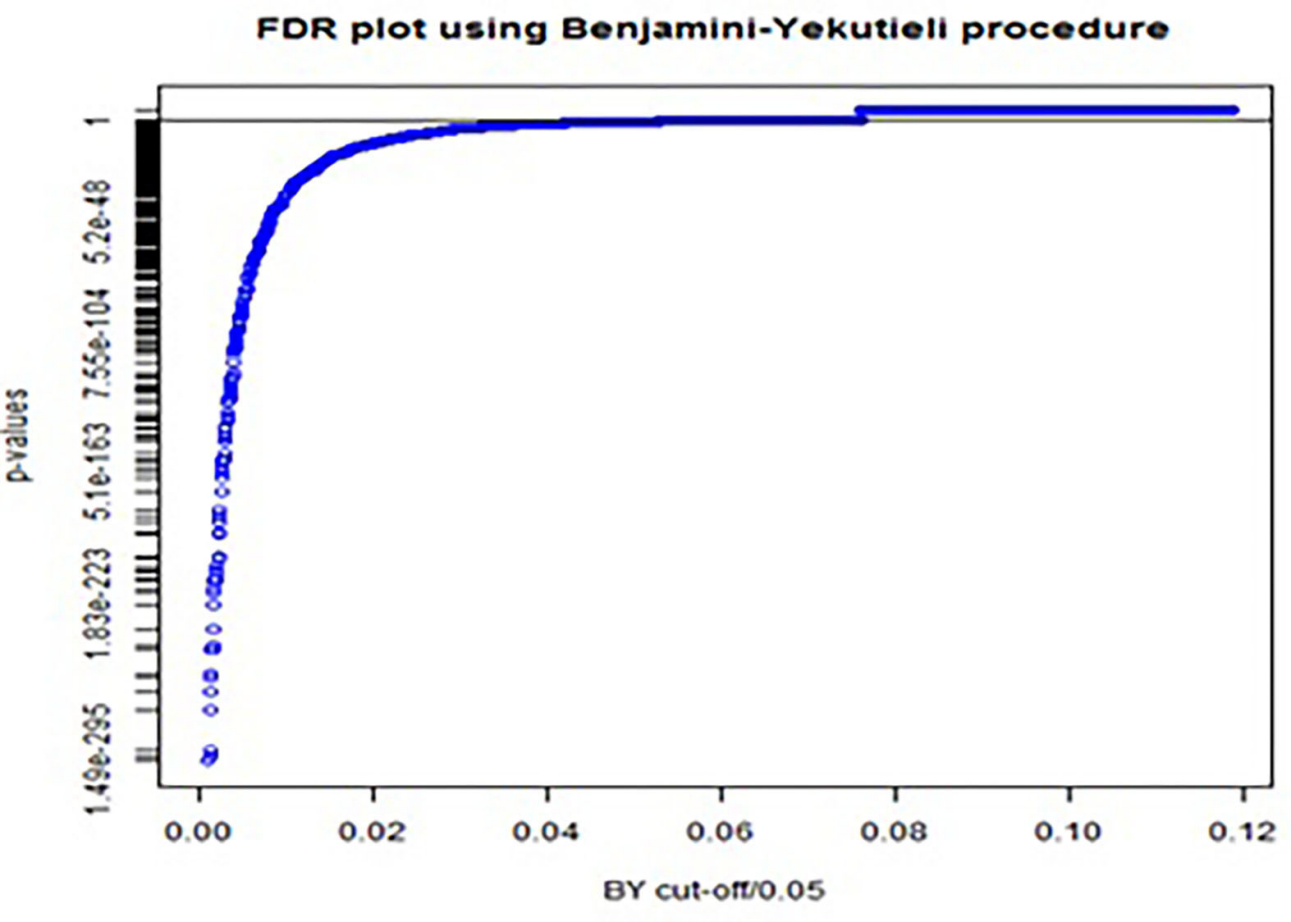
FDR plot using Benjamini-Yekutieli procedure for adjusting *p*-values in multiple testing with FDR controlled at 5%.

In total, 2,600 McNemar tests were conducted on the training data set. The conservative Bonferroni procedure captured 630 out of 2,600 as significant codes while controlling FWER at 5%, whereas the BY procedure captured 775 significant ICD-10 codes when controlling FDR. Note that, all 630 codes captured by the Bonferroni procedure were also captured by BY procedure. The *p*-values for the BY procedure, with FDR controlled at 5%, were plotted (Figure 2). We can get a visual impression of the proportion of significant ICD-10 codes. The solid black line represents the cut-off value, indicating that all *p*-values below the cut-off value are significant. Odds ratios (ORs) and their respective 95% confidence intervals (CIs) were calculated for the 775 codes, of which 684 had OR>1.

The 684 codes identified from the training data set were retested using the validation data set, and the BY procedure captured 584 of them as significant. Finally, ORs for these 584 codes were calculated using the testing data set. Top six significant ICD-10 codes with the highest ORs are listed in Table 2. These codes were found to be associated with TBI in the existing epidemiologic literature. The remaining ICD-10 codes relevant to TBI are reported in a follow-up work (Mollayeva et al., 2019).

**Table 2:** Top 6 significant ICD-10 codes, with highest OR, associated with TBI.

**ICD-10 Code**	**Description**	**OR (LCL, UCL)**	**Comments**	**Supporting citations**
Y91	Evidence of alcohol involvement determined by level of intoxication	60 (14.83, 242.69)	Associated with severity and occurrence of TBI, prevalent among trauma and brain injury patients	(Kraus et al., 1989; Rivara et al., 1993; S Tate et al., 1999; Cunningham et al., 2002; Colantonio et al., 2011; Thompson et al., 2012)
F07	Personality and behavioral disorders due to known physiological condition	56.67 (18.09, 177.45)	PCS (F07.2) have been found to be associated with TBI post injury	(Boake et al., 2005; Kashluba et al., 2006; Yang et al., 2007; Halbauer et al., 2009; Williams et al., 2010; Colantonio et al., 2011; Thompson et al., 2012)
Y00	Assault by blunt object	22 (9.71, 49.86)	Prevalent as a prior event among TBI patients across several countries	(Nell and Brown, 1991; Stiell et al., 2001; Langlois et al., 2003; Hyder et al., 2007; Fernandes and Silva, 2013)
Y09	Assault by unspecified means	18.57 (8.69, 39.73)	Cause of head injury and head trauma across different countries	(Kleiven et al., 2003; Jamieson et al., 2008; Parks et al., 2012)
S27	Injury of other and unspecified intrathoracic organs	17.6 (7.15, 43.34)	Blast-induced thoracic mechanism results in TBI	(Courtney and Courtney, 2009, 2011)
Y04	Assault by bodily force	12.04 (10.06, 14.41)	Identified as an external cause of TBI in different countries	(Colantonio et al., 2010; Fernandes and Silva, 2013; Hamill et al., 2015; Cheng et al., 2017)

OR, odds ratio; LCL, lower control limit; UCL, upper control limit.

As this is a pedagogical article, we have provided a sample data set (Supplementary material) and a sample R code (Supplementary material), which readers may use to become more acquainted with the steps employed in this study. The data set and R code that have been provided, however, are solely for illustration purposes. The sample dataset contains 1,239 ICD-10 codes, corresponding McNemar test statistic values, unadjusted *p*-values, ORs, and 95% CIs of the ORs. Once the R code is run using this data set, one will obtain 781 significant ICD-10 codes out of 1,239 codes provided in the data set and 686 relevant codes out of 781. This sample code illustrates the use of the BY method, which is the prime focus of this study. The dataset can be regarded as a training data set, and the same steps can be repeated on these 686 codes using the validation data set. Finally, the testing data set can be used for the final reporting of ORs and 95% CIs. Please note that this is only a sample data and not the complete data set used to obtain the results in this or the follow-up works (Mollayeva et al., 2019, 2022).

Statistical analyses in this article were done using the statistical software **R 3.3.0 (**R Foundation for Statistical Computing; www.r-project.org) and **SAS 9.3** (SAS software: version 9.3, SAS Inc., Cary, NC).

## Discussion

We implemented two MTPs to assess associations between 2,600 ICD-10 codes and TBI in this research. A total of 684 relevant codes captured in this study have been reported and used for further analysis in the follow-up studies (Mollayeva et al., 2019, 2022). We have successfully shown that the developed proof-of-concept worked with data and that supportive evidence on the association of TBI with the top six codes was found in contemporary literature. Although the proof-of-concept implementation only shows one case example, TBI, the methods can be reused for different population data.

The BY procedure implemented in this study initially captured 775 ICD-10 codes as significant codes. However, we would like to highlight that, of these 775 codes, we considered only codes with OR>1 as codes relevant to TBI, which led to only 684 relevant codes. This is because our reference group, unlike any control group, does not consist of healthy individuals. Therefore, comorbid conditions, such as female infertility, were observed to be highly correlated with TBI with a *p*-value of 3.3 × 10^−16^ and an OR of 0.072, appearing to be protective of a TBI event. This interpretation is, however, misleading. Such a strong negative correlation between female infertility and TBI is because there were many patients in our data set diagnosed with female infertility who did not have TBI, and this has no link with the association between female infertility and TBI. TBI severity or concussions were unspecified in the data set. The observed association might be owing to care-seeking behaviors in concussive injury, few females sought care after mild TBI/concussion as compared to females without TBI.

This phenomenon of strong negative correlation was observed for a few other ICD-10 codes as well. This is also termed as collider effect or sampling bias and has been illustrated in detail in Carvajal-Rodríguez (2018). Hence, we removed significant codes with OR < 1. As is evident, the top 2 codes with the highest ORs captured in this study are alcohol involvement and neurological disorders. These comorbidities were observed to be present in TBI and acquired brain injury (ABI) patients in other studies as well (Colantonio et al., 2011; Thompson et al., 2012). Interesting findings about contrasting profiles of TBI vs. non-TBI patients have been presented in this study (Colantonio et al., 2011).

Some limitations are related to the use of health-administrative data in our research. Typically, acute care data provided by ICES are reported to be accurate and undergo quality assurance on a regular basis^1^ (Cole et al., 2010). While we used validated algorithms to define TBI, each comorbidity captured within the emergency department and acute care visits is also characterized by certain sensitivity and specificity, resulting in possible misclassification, although the misclassification, if present, would refer to both TBI cohort and the reference cohort. Methods for adjusting for misclassification exist; however, it was not within the scope of this study to perform the adjustments. Further studies are required to address this limitation.

### What this study adds

The purpose of using MTPs in a study such as this is dimension reduction. Big health-administrative data sets contain information on multiple correlated comorbidities. To study associations of these comorbidities with the condition of interest, thousands of tests need to be performed for each comorbidity simultaneously, which would lead to a multiplicity problem, leading to a huge number of false rejections. However, a screening approach using MT adjustments to capture relevant codes for correlated tests considerably reduces the dimension of the data set to a smaller space, consequently reducing the multiplicity. This is an essential step before any subsequent analysis is done, as it improves the predictive power of the analysis.

## Conclusion and future directions

In this study, using knowledge translations from other fields of high-dimensional data, such as statistical genetics, we developed a statistical approach and applied it for analyzing decade-long health-administrative data of patients with TBI and individually matched on sex, age, socio-economic status and neighborhood, with non-TBI patients. We illustrated the utility of classical and modern statistical tools for assessing comorbidity in big health-administrative data sets, which can be applied to any extensive health-administrative data set to study associations between comorbidities represented by ICD-10 codes and any condition of interest.

### Future directions

Assuming a general dependence structure among comorbidities, we used the Benjamini-Yekutieli method to adjust multiplicity in correlated tests. Other more powerful methods for FDR control based on resampling can be used as well; however, implementing such methods on high-dimensional data sets will require huge computational time, system memory allocation and state-of-the-art computational facilities.

We would like to highlight that, in this study, we have done the analysis only on 2,600 ICD-10 codes, due to time and system memory constraints; however, the analysis can be easily extended to more specific 26,000 ICD-10 codes. One can be as granular as one wishes for ICD-10 codes, subject to system and time constraints.

Future work can also consider assimilating data from different populations/strata and implementing more modern FDR controlling procedures such as stratified FDR to give us an overall picture. Please note that the regular FDR is a weighted average of the stratified FDR, and hence, the latter is computationally intensive (Sun et al., 2006).

Considerable work is required for the automation of the process outlined in this article using autonomic computing, which is the future of the next-generation computing (Gill et al., 2022). The benefit of having such AI-based automated computing system is the low cost incurred in implementing and maintaining them. Anomaly detection, record-keeping, data organization, and cleaning can be made up to date with real-time inputs and computation (Gill et al., 2022). Such methods can be easily implemented across several domains of applications including mining of health-administrative data and would be an interesting future research that can benefit the field of public health.

## Data availability statement

The data analyzed in this study is subject to the following licenses/restrictions: The datasets generated during and/or analyzed during the current study are available in the ICES repository, under accession DAS 2016-257(2018 0970 084 000). Data sharing agreements prohibit ICES from making the datasets publicly available, however access may be granted to those who meet pre-specified criteria for confidential access. This study made use of de-identified data from the ICES Data Repository, which is managed by the Institute for Clinical Evaluative Sciences with support from its funders and partners: Canada's Strategy for Patient-Oriented Research (SPOR), the Ontario SPOR Support Unit, the Canadian Institutes of Health Research and the Government of Ontario. The opinions, results and conclusions reported are those of the authors. No endorsement by ICES or any of its funders or partners is intended or should be inferred. Parts of this material are based on data and information compiled and provided by the Canadian Institute for Health Information (CIHI). However, the analyses, conclusions, opinions and statements expressed herein are those of the author, and not necessarily those of CIHI. The full dataset creation plan and underlying analytic code are available from the authors upon request, understanding that the computer programs may rely upon coding templates or macros that are unique to ICES and are therefore either inaccessible or may require modification. A sample dataset (Supplementary material) with the ICD-10 codes and p-values from the McNemar test statistics on which the MTPs were used are attached as Supplementary material to this manuscript. A sample R code (Supplementary material) is also provided for the readers to run the code and obtain outputs from the data. Requests to access these datasets should be directed to www.ices.on.ca/DAS, http://www.ices.on.ca/DAS.

## Ethics statement

The studies involving human participants were reviewed and approved by the Ethics Committees at the Clinical (Toronto Rehabilitation Institute-University Health Network) and Academic (University of Toronto) Institutions. Written informed consent from the patient/participants or patients/participants' legal guardian/next of kin was not required to participate in this study in accordance with the national legislation and the institutional requirements.

## Author contributions

SJ carried out the analysis with support from ME, MS, and VC and drafted the manuscript. MS cleaned and prepared the data set for analysis, and developed and implemented codes for some supplementary analysis with support from ME. AC, VC, and TM conceived the original concept and initiated the work. ME designed and optimized statistical analyses for this work. He provided major feedback on the manuscript, and supervised and mentored during the analysis and manuscript development stage. SJ is the first author and ME is the senior author. All authors provided their feedback on the manuscript and the analysis.

## Funding

This study was supported by the Eunice Kennedy Shriver National Institute of Child Health & Human Development of the National Institutes of Health (NIH) [Award No. R21HD089106] and the National Institute of Neurological Disorders and Stroke of the National Institutes of Health under Award Number R01NS117921. AC was funded by the Canadian Institutes of Health Research (CIHR) Chair in Gender, Work and Health [Grant No. CGW-126580], and TM was supported by Canada Research Chair in Neurological Disorders and Brain Health and the Alzheimer's Association Grant [AARF-16-442937]. Please note that the content of the article is solely the responsibility of the authors and does not necessarily represent the official views of the NIH. This work was funded in part by the Canada Research Chairs Programme. The funders had no role in the study design, data collection, decision to publish or preparation of the manuscript.

## Conflict of interest

The authors declare that the research was conducted in the absence of any commercial or financial relationships that could be construed as a potential conflict of interest.

## Publisher's note

All claims expressed in this article are solely those of the authors and do not necessarily represent those of their affiliated organizations, or those of the publisher, the editors and the reviewers. Any product that may be evaluated in this article, or claim that may be made by its manufacturer, is not guaranteed or endorsed by the publisher.

## Footnotes

^1^Available online at: https://www.cihi.ca/en/topics/emergency-and-ambulatory-care#_Databases_and_Data.

## Abbreviations

MTP: multiple testing procedure
MT: multiple testing
FDR: false discovery rate
FWER: family-wise error rate
ICD-10: International Statistical Classification of Diseases and Related Health Problems (10^th^ revision)
TBI: traumatic brain injury
ICES: Institute for Clinical Evaluative Sciences.
