# Supplementary material for: Application of multiple testing procedures for identifying relevant comorbidities, from a large set, in traumatic brain injury for research applications utilizing big health-administrative data
Source: Front Big Data. 2022 Sep 28;5:793606. doi: 10.3389/fdata.2022.793606 (PMC9563390; doi:10.3389/fdata.2022.793606)
Supplement: Supplementary file 2 [file Table_1.docx]

**Table 2: Top 6 significant ICD-10 codes, with highest OR, associated with TBI**

| ICD-10 Code | Description | OR (LCL, UCL) | Comments | Supporting citations |
| --- | --- | --- | --- | --- |
| Y91 | Evidence of alcohol involvement determined by level of intoxication | 60 (14.83, 242.69) | Associated with severity and occurrence of TBI, prevalent among trauma and brain injury patients | Cunningham et al (33), Tate et al (34), Rivara et al (35), Kraus et al (36), Colantonio et al (37), Thompson et al (38) |
| F07 | Personality and behavioral disorders due to known physiological condition | 56.67 (18.09, 177.45) | PCS (F07.2) have been found to be associated with TBI post injury | Boake et al (39), Yang et al (40), Kashluba et al (41), Halbauer et al (42), William et al (43), Colantonio et al (37), Thompson et al (38) |
| Y00 | Assault by blunt object | 22 (9.71, 49.86) | Prevalent as a prior event among TBI patients across several countries | Langlois et al (44), Stiell et al (45), Fernandes and Silva (46), Hyder et al (47), Nell and Brown (48) |
| Y09 | Assault by unspecified means | 18.57 (8.69, 39.73) | Cause of head injury and head trauma across different countries | Kleiven et al (49), Jamieson et al (50), Parks et al (51) |
| S27 | Injury of other and unspecified intrathoracic organs | 17.6 (7.15, 43.34) | Blast-induced thoracic mechanism results in TBI | Courtney and Courtney (52,53) |
| Y04 | Assault by bodily force | 12.04 (10.06, 14.41) | Identified as an external cause of TBI in different countries | Hamil et al (54), Cheng et al (55), Colantonio et al (56), Fernandes and Silva (46) |

Abbreviations: OR = odds ratio; LCL= lower control limit; UCL= upper control limit
