# Supplementary material for: Application of multiple testing procedures for identifying relevant comorbidities, from a large set, in traumatic brain injury for research applications utilizing big health-administrative data
Source: Front Big Data. 2022 Sep 28;5:793606. doi: 10.3389/fdata.2022.793606 (PMC9563390; doi:10.3389/fdata.2022.793606)
Supplement: Supplementary file 3 [file Table_2.DOCX]

setwd(<folder path>) # setting the desired folder as our working directory

# reading the data in R

train_data=read.csv("Additional File 1.csv")

train_data=train_data[,-4]

# taking a look at the first few rows of the data to get a feel of the data

head(train_data)

# adjusting the p-values using BY method , this code can be repeated with method="bonferroni"

pv_train=train_data$pvalue

# storing the new adjusted p values in pv_adj_train

pv_adj_train=round(p.adjust(pv_train,method="BY"),9)

# here my objective is to create a new column in the data called sig_adj which will declare significance by

# looking at the adjusted p-values, then we can compare sig_adj with the column significance as a check

n1=nrow(train_data)

sig_adj=rep(".",n1)

# replacing the NAs in the adjusted p-values

pv_adj_train[is.na(pv_adj_train)] = 999

# determining significance by looking at the adjusted p-values

for(i in 1:n1)

{

if(pv_adj_train[i]<0.05){sig_adj[i]="*"}

}

# inserting this new column of significance into the dataframe

train_data_adj=cbind(train_data,pv_adj_train,sig_adj)

head(train_data_adj)

tail(train_data_adj)

# count the no. of significances

sum(sig_adj=="*")

# counting the no of relevant codes (significant and OR > 1)

train_data_relevant=subset(train_data_adj, ORest_pre>1)

sum(train_data_relevant$sig_adj=="*")
