# Supplementary material for: Application of multiple testing procedures for identifying relevant comorbidities, from a large set, in traumatic brain injury for research applications utilizing big health-administrative data
Source: Front Big Data. 2022 Sep 28;5:793606. doi: 10.3389/fdata.2022.793606 (PMC9563390; doi:10.3389/fdata.2022.793606)
Supplement: Supplementary file 4 [file Table_4.docx]

**Appendix**

A brief review of the theory of multiple testing and the FDR

The underlying principle of this paper is that there are many possible variables which are associated with an outcome of interest. The goal is to individually test each variable one at a time to get a reduced set of ‘important’ variables. One can then use this smaller group of variables for further analyses.

Going back to basic principles of statistical inference, the general idea of hypothesis testing is to see if an effect is important by assuming that the effect was not important (the null hypothesis) and then calculating a test statistic from the observed data. One then looks at the probability, under the null hypothesis, of encountering a value as large as the observed test statistic or greater. If this probability is bigger than some number, usually called the α level, then one would say that the null hypothesis is rejected and the effect is statistically significant. Otherwise, one would say that the null hypothesis is not rejected. Therefore, there are two types of errors, one either accepts the null hypothesis when it is actually false (Type II error) or rejects the null hypothesis when it is true (Type I error). Note that when one rejects the null hypothesis, one often says that the effect is statistically significant and one provisionally considers that the effect is not due to chance alone.

False rejection of the null hypothesis happens by chance only α percent of times (say 5%). Therefore, the issue is to spot these “false rejections”. With an α level of .05, there is a 5% chance that the results are just random, if there are no “effects” in the model/data/study. One problem with the above set up is that if one performs many tests, then the chance of seeing a rare event is more probable. So, with multiple tests, the chance of falsely rejecting one null hypothesis increases. If one does 20 tests, even if there is nothing going on, with an α of 5%, one would expect one false rejection for every 20 tests. In general, with *m* tests and no true effects, one would expect to find *m**α false rejections. There is an argument to control for these random results (12–16). An older way to do this is to control for the FWER. The method advocated here is to limit the FDR.

In any hypothesis testing problem, probability of Type I error, is controlled at significance level *α*. For a single hypothesis test, the level of significance is called individual level or individual error rate. Note that, when the probability of Type I error is *α*, the probability of not rejecting a true null hypothesis is $(1- \alpha)$. Now, instead of a single null hypothesis, if we test *m* independent hypotheses, the probability of rejecting at least one of the *m* hypotheses, when all the *m* null hypotheses are true, is $[1-\left( 1- \alpha\right)^{m}]$. This is called the global level or family-wise error rate (FWER). It is the probability of making at least one Type I error. For a family of *m* independent tests, significance level is controlled at global level $1-{(1- \alpha)}^{m}$ instead of at individual level *α* (12).

Olive Jean Dunn (5) proposed that for *m* independent tests, of which *m_0_* are true, the rejection criteria for each of the tests be $p_{i} \leq\alpha/m$, where $p_{i}’s \left( i=1,2, \ldots,m \right)$are the individual p-values. This is popularly called the Bonferroni correction and it controls the FWER at *α.* The Bonferroni correction does not depend on the number of true null hypotheses, rather depends only on the number of hypotheses being tested and each test is conducted at a much lower level $\alpha/m$ instead of *α* (5,49).

Although the Bonferroni correction protects against false positives, it was later discovered that the correction substantially reduces power to detect true signals when the number of tests is large, and it is very conservative (4,7–9). Other more powerful FWER controlling procedures were later developed, however, they too have substantially low power as the number of multiple tests increase (49-51).

However, as stated before, scientists are more interested in detecting true signals rather than just preventing a large number of false positives by controlling the FWER. Benjamini and Hochberg (1995) (11) suggested controlling the FDR, which is the expected proportion of false discoveries, a discovery being a rejected hypothesis or in other words a ‘signal’, as already stated.

To understand the difference between FWER and FDR, consider the table below which summarizes the results of *m* different tests. The rows represent test results where the test rejects the null hypothesis or not. When one rejects the null hypothesis, then one might say that the test showed a statistically significant result. Also, one might say that the test showed a “signal” of an effect. The columns in the table represent the true state of nature. When the alternative hypothesis is true, and the test result is significant, one might consider calling this a “true signal”.

Let us consider the following setup for ‘*m’* multiple tests. Let $H_{1} , H_{2} , \ldots, H_{m}$be the ‘*m’* null hypotheses of which *m_0_* are true. Let *R* be the number of discoveries of which *S* are true and *V* are false (Type I error or false positives), and let *U* be the number of true negatives and *T* be the number of false negatives (Type II error).

| Truth  Decision | Null hypothesis is true | Alternative hypothesis is true | Total |
| --- | --- | --- | --- |
| Test is significant  (signal) | *V* | *S* | *R* |
| Test is non-significant  (not a signal) | *U* | *T* | *m-R* |
| Total | *m_0_* | *m- m_0_* | *m* |

Evidently, *R* is observable, *m* is known and *V, S, U, T, m_0_* are unknown. That is, we would know the number of individual tests where we reject the null hypothesis (*R*) and the number of tests performed (*m*). Perhaps the most interesting number in the table is *V* which is the number of tests where a true null hypothesis is rejected by the test. That is, this is the number of tests that are falsely declared statistically significant. The purpose of FWER is to control the probability that *V* is one or more at some set value α.

When one is looking at hundreds or millions of tests, then this might be a bit extreme. Alternatively, we look at the FDR. Since, the proportion of false discoveries is defined as *Q=V/R* then FDR=*E(Q).* Therefore, the FWER looks at guarding against any *V* events, that is, control the probability that *V =0*. In contrast, the thought behind FDR is that one controls the rate *V/R*. That is, there are *R* tests which reject the null hypothesis, which is when the investigator says that they are statistically significant, or would consider them to be signals. Therefore, with a set of tests where one suspects a signal, one wants to control for the number of “false signals”. Note that, FDR=*E(Q)* only when *R>0* and when *R=0*, then *Q=0*, and consequently FDR=*0* (52).

It is essential to bring to the attention of the readers that, controlling FDR at level *α*, does not automatically guarantee that the remaining $100(1- \alpha)\%$ of the discoveries are true discoveries. However, if the data contains some true discoveries then the Benjamini-Hochberg procedure ensures to capture around $100(1- \alpha)\%$ true discoveries (53).

Benjamini and Hochberg (11) proposed a linear step-up procedure that controls FDR at level *α*, which we outline here. Let the ordered p-values for the ‘*m’* null hypotheses be $P_{(1)} \leq P_{(2)} \leq\ldots\leq P_{(m)}$ and the corresponding null hypotheses be $H_{(1)} \leq H_{(2)} \leq\ldots\leq H_{(m)}$. To control FDR at level *α*, each ordered p-value $P_{\left( i \right)}\left( i=1,2, \ldots, m \right)$is compared with the cut-off $\alpha.i/m$, and the highest rank ‘*k’* is identified such that$k=max\{i: P_{(i)}\leq\alpha.i/m\}$*.* Then the hypotheses $H_{(1)} \leq H_{(2)} \leq\ldots\leq H_{(k)}$ are declared significant. Benjamini and Hochberg (11) showed that this procedure actually controls the FDR at $\alpha\frac{m_{0}}{m}\leq\alpha$. This is true for both independent tests as well as positively correlated tests. There is some literature available on estimating $m_{0}$ (54-57). Benjamini and Hochberg (11) also showed that FDR ≤ FWER and equality holds when all ‘*m’* null hypotheses are true ie, when $m_{0}=m$. Consequently, when all *‘m’* null hypotheses are true, controlling for FDR also in turn controls FWER. However, note that, for $m_{0}<m$ this procedure does not control FWER (6,11,21,52) .

Due to the drastically reduced cost of experiments, and better and improved technology, especially in genetics where number of tests is too large, typically in the billions, FDR is more relevant than FWER, since geneticists want to capture all true signals. Therefore, like most other scientific fields, geneticists are more interested in controlling the proportion of false rejections rather than just maintaining a low probability of at least one false rejection, since few erroneous rejections will not affect the conclusions as long as their proportion is controlled at a desired level. In summary, FDR is emerging as a modern and more appealing screening tool for multi-stage analyses (4,6,7).

Few studies have conducted extensive simulations to compare different FWER controlling procedures with FDR controlling procedures, in terms of power and control of Type I error rates. In general, it was found that FDR controlling procedures are uniformly more powerful than FWER controlling procedures (6). This study also observes that the adjusted p-values for FDR controlling procedures are larger than the true, guaranteeing FDR control, and are much closer to true FDR than the adjusted p-values from FWER controlling procedures. However, this study did not present any result comparing the Benjamini-Hochberg procedure with the Bonferroni method, in particular. Another study (9) compared these two methods using simulation studies and concluded that the former has substantially more power and this increase in power is uniform with increasing number of tests. Nevertheless, Sun et. al. (7) remarks that a power comparison between the two methods is inappropriate since they control Type I error rate at different levels.

The Benjamini and Hochberg (11) procedure can also be formulated in an alternative manner. Instead of comparing each p-value with a separate cut-off, an alternative measure, called the adjusted p-values, $P_{(j)}^{*}=\begin{matrix} min \\ j\leq i \end{matrix}\{P_{\left( i \right)} . \frac{m}{i}\}$ , can be computed and the corresponding null hypothesis be declared significant if $P_{(j)}^{*}$*≤ α*. This procedure is easier to implement for practitioners as it is analogous to the usual p-value method for a single hypothesis.

As mentioned before, the Benjamini-Hochberg (11) procedure controls FDR at *α* only for independent and positively correlated tests. For a more general dependency structure, Benjamini and Yekutieli (21) demonstrated that comparing the ordered p-values $P_{\left( i \right)}\left( i=1,2, \ldots, m \right)$with the cut-off $\frac{\alpha}{\sum_{i=1}^{m} \frac{1}{i}} \frac{i}{m}$ instead of at $\alpha*i/m$ and then rejecting $H_{(1)} \leq H_{(2)} \leq\ldots\leq H_{(k)}$ where $k=max\{i:P_{\left( i \right)}\leq\frac{\alpha}{\sum_{i=1}^{m} \frac{1}{i}} \frac{i}{m}\}$, controls FDR at level *α*. In fact, this procedure controls FDR at a much lower level $\alpha*\frac{m_{0}}{m}$ (6,21). As is evident, the only difference between the Benjamini-Hochberg and the Benjamini-Yekutieli methods is in the calculation of the cut-off. aa
